# Effects of Exenatide vs. Metformin on endothelial function in obese patients with pre-diabetes: a randomized trial

**DOI:** 10.1186/1475-2840-11-64

**Published:** 2012-06-08

**Authors:** Aaron S Kelly, Richard M Bergenstal, J Michael Gonzalez-Campoy, Harold Katz, Alan J Bank

**Affiliations:** 1 Department of Pediatrics, University of Minnesota Medical School, Minneapolis, MN, USA; 2 International Diabetes Center at Park Nicollet, St. Louis Park, MN, USA; 3 Minnesota Center for Obesity, Metabolism and Endocrinology, Eagan, MN, USA; 4 Allina Hospitals and Clinics, St. Paul, MN, USA; 5 United Heart and Vascular Clinic, St. Paul, MN, USA

**Keywords:** Exenatide, Metformin, Endothelial function, Obesity, Pre-diabetes

## Abstract

**Background:**

Glucagon like peptide-1 (GLP-1) receptor agonist treatment may improve endothelial function via direct and indirect mechanisms. We compared the acute and chronic effects of the GLP-1 receptor agonist exenatide vs. metformin on endothelial function in patients with obesity and pre-diabetes.

**Methods:**

We performed a randomized, open-label, clinical trial in 50 non-diabetic individuals (mean age 58.5 ± 10.0; 38 females) with abdominal obesity and either impaired fasting glucose, elevated HbA1c, or impaired glucose tolerance (IGT) who were randomized to receive 3-months of exenatide or metformin. Microvascular endothelial function, assessed by digital reactive hyperemia (reactive hyperemic index: RHI), C-reactive protein (CRP), circulating oxidized LDL (oxLDL), and vascular cell adhesion molecule-1 (VCAM-1) were measured at baseline and 3-months. Seven subjects with IGT participated in a sub-study comparing the effects of pre-administration of exenatide and metformin on postprandial endothelial function.

**Results:**

There were no differences for the change in RHI (Δ exenatide: 0.01 ± 0.68 vs. Δ metformin: -0.17 ± 0.72, P = 0.348), CRP, oxLDL, or VCAM-1 between exenatide and metformin treatment. Triglycerides were reduced more with exenatide compared to metformin (Δ exenatide: -25.5 ± 45.7 mg/dL vs. Δ metformin: -2.9 ± 22.8 mg/dL, P = 0.032). In the sub-study, there was no difference in postprandial RHI between exenatide and metformin.

**Conclusions:**

Three months of exenatide therapy had similar effects on microvascular endothelial function, markers of inflammation, oxidative stress, and vascular activation, as metformin, in patients with obesity and pre-diabetes.

**Clinical trials registration:**

This study is registered on http://www.clinicaltrials.gov/: NCT00546728

## Background

The term pre-diabetes is used to describe individuals with either impaired fasting glucose (IFG), elevated glycosylated hemoglobin (HbA1c), or impaired glucose tolerance (IGT). In parallel with the increase in the prevalence of obesity, the number of individuals with pre-diabetes is growing rapidly [1]. In addition to being predictive of type 2 diabetes mellitus (T2DM), pre-diabetes is associated with increased risk for developing cardiovascular disease (CVD) [2,3]. The preferred treatment approach for pre-diabetes is lifestyle modification emphasizing healthier eating habits and increased levels of physical activity, ideally leading to weight-loss. However, drug therapy is also used to treat pre-diabetes with the goal of preventing the onset of frank T2DM. Most prominently, metformin and the peroxisome proliferator-activated receptor-γ agonists pioglitazone and rosiglitazone have been shown to attenuate the transition from pre-diabetes to T2DM [4-6]. Metformin is perhaps the most widely-used medication to treat pre-diabetes because of its generally well-accepted safety profile and tendency to help patients maintain or reduce body weight [5].

The potential cardio-protective effects of medications should be an important consideration when choosing drug therapy for pre-diabetes given its association with CVD. Medications that attenuate postprandial glucose spikes may be particularly attractive since these glucose excursions are associated with endothelial dysfunction, inflammation, oxidative stress, and atherosclerosis in individuals with pre-diabetes [7-12]. In this regard, the glucagon-like peptide-1 (GLP-1) receptor agonist class may be an ideal candidate due to its primary mechanisms of action: reduction of postprandial glucose via increasing insulin secretion, decreasing glucagon secretion, and slowing gastric emptying [13], which leads to improved chronic glycemic control even when used in combination with other diabetes medications [14,15]. Moreover, evidence suggests that GLP-1 may act directly on the endothelium to improve endothelial function and inhibit atherosclerosis [16-21] and may have additional beneficial cardiovascular effects [22,23].

The effect of GLP-1 receptor agonist treatment on endothelial function has not been well-described in humans. Therefore, we performed a randomized, head-to-head clinical trial comparing the acute and chronic effects of the GLP-1 receptor agonist exenatide vs. metformin on microvascular endothelial function in patients with obesity and either IFG, elevated HbA1c, or IGT. We chose metformin as the comparator since it is generally viewed as a first-line drug therapy in the context of pre-diabetes and has a strong evidence-base supporting its use in this condition [5].

## Methods

### Patient population

Fifty non-diabetic individuals (waist circumference ≥102 cm for men and ≥88 cm for women) with abdominal obesity and either IFG (fasting glucose ≥100 mg/dL), elevated HbA1c (≥5.7%), or IGT (2-hour glucose ≥140 mg/dL), were enrolled at two sites from 2007–2010: the United Heart and Vascular Clinic and the International Diabetes Center at Park Nicollet. Patients were excluded if they had T2DM, were not on a stable (≥ 1-month) cardiovascular medication regimen (e.g., anti-hypertensive therapy, statins, etc.), had used medications for glycemic control within 1-month, had previous bariatric surgery, or had a history of severe gastrointestinal disease, unstable angina or heart failure. Patients were recruited from local medical clinics and through advertisements. The study protocol was approved by the institutional review board at Copernicus Group IRB, the University of Minnesota, and the International Diabetes Center at Park Nicollet and written consent was obtained from all participants.

### Study design

We performed a 3-month, randomized (1:1), open-label, head-to-head (exenatide vs. metformin) clinical trial. Following baseline testing, patients were randomly assigned (blocked randomization to ensure an equal number per treatment group by site) to treatment with exenatide or metformin for 3-months. Measurements of study variables were made at baseline and 3-months. All testing was performed in the morning after patients had been fasting for at least 12 hours. A sub-study was performed in seven patients with IGT to evaluate the acute postprandial (acute glucose challenge) effects of exenatide and metformin on endothelial function. All participants in the sub-study underwent testing with pre-administration of both medications and served as their own control. The sub-study included two extra visits, each requiring a standard oral glucose tolerance test (OGTT) with pre-administration of exenatide (10 mcg, 30-minutes prior to glucose ingestion) during one visit and metformin (1000 mg, 60-minutes prior to glucose ingestion) during the other visit. The order of the conditions (exenatide and metformin) was randomized. The control condition (no drug pre-administration to establish a baseline) was performed during a third visit (the main-study baseline visit since all sub-study subjects also participated in the main study).

### Exenatide and metformin treatment protocol

Exenatide was initiated at a dose of 5 mcg, BID for 1-month and up-titrated to 10 mcg, BID for the remaining 2-months. Metformin was initiated at a dose of 500 mg, BID for 1-month and up-titrated to 1000 mg, BID for the remaining 2-months. Individuals who did not tolerate the higher doses of exenatide and metformin were allowed to continue the study on the lower doses. Study medications were withheld on the mornings of the study visits (except for sub-study visits).

### Measurement of clinical variables

Height and weight were obtained using a standard stadiometer and electronic scale, respectively. Body mass index (BMI) was calculated as weight in kilograms divided by height in meters squared. Waist circumference was obtained at end-expiration and measured midway between the base of the rib cage and the superior iliac crest. The study was performed at two sites, each with different technologies to measure body fat. At one site, total body fat was measured with dual energy x-ray absorptiometry (N = 35) (Hologic Discovery, Hologic, Inc., Bedford, MA, USA), and at the other, abdominal visceral/subcutaneous fat was measured by computed tomography using the average of slices obtained at L1/L2 and L4/L5 (N = 14) (Siemens SOMATOM Definition, Siemens Healthcare, Malvern, PA, USA). Sitting blood pressure measurements were obtained manually on the same arm using the same cuff size and equipment after the patient had been resting quietly for 10 minutes. Fasting lipid profile, glucose, and insulin assays were conducted with standard procedures by Quest Diagnostics (Minneapolis, Minnesota). Homeostasis model assessment of insulin resistance (HOMA-IR), a surrogate measure of insulin resistance, was calculated using previously described methods [24].

### Plasma biomarkers

Blood plasma for biomarker analysis was stored at −80°C until the end of the study at which time all samples were assayed together. C-reactive protein (CRP) (ALPCO Diagnostics, Salem, NH, USA), circulating oxidized LDL (oxLDL) (Mercodia, Inc., Winston-Salem, NC, USA), and vascular cell adhesion molecule-1 (VCAM-1) (R&D Systems, Inc., Minneapolis, MN, USA) were measured by ELISA in the University of Minnesota Cytokine Reference Laboratory (CLIA licensed).

### Endothelial function assessment

Microvascular endothelial function was measured by digital reactive hyperemia (EndoPAT 2000, Itamar Medical, Caesarea, Israel), an operator-independent method of quantifying endothelial function. Digital reactive hyperemia is nitric oxide-dependent [25], associated with coronary artery blood flow [26] and multiple cardiovascular risk factors [27], and independently predicts future cardiovascular events [28]. Following 10 minutes of quiet rest in the supine position, finger probes were placed on the index fingers of both hands to measure baseline and reactive hyperemic pulse amplitude. The probes applied a uniform pressure (10 mmHg less than DBP) on the fingers, which allowed for the detection of small pulse volume changes throughout the cardiac cycle. Following the collection of five minutes of baseline data, a blood pressure cuff on the upper arm was inflated to a suprasystolic level for five minutes. Following cuff release, the change in pulse amplitude during reactive hyperemia was measured for five minutes. The ratio of the hyperemic and the baseline pulse amplitude (corrected for the same ratio on the control finger) was calculated and expressed as the reactive hyperemic index (RHI). For the sub-study, RHI was measured at baseline (pre-OGTT) and 1- and 2-hours during the OGTT.

### Statistical analysis

Baseline variables were compared between the exenatide and metformin groups using independent samples t-tests. For the main study, 2 X 2 (group X time) analysis of variance (ANOVA) was used to compare changes in variables between groups before and after the 3-month treatment period. The ANOVA interaction term was the pre-specified analysis of interest. For descriptive purposes, within-group treatment effects were analyzed with paired samples t-tests. For the sub-study, the area under the curve (AUC) for glucose, insulin, and RHI was calculated for each of the three conditions (control, exenatide, and metformin) and compared by ANOVA. An alpha value of 0.05 was used to determine statistical significance. Data are presented as mean ± standard deviation. GraphPad Prism version 5.0 (GraphPad Software, Inc., La Jolla, CA, USA) was used for statistical analyses.

## Results

### Main study

Fifty patients with abdominal obesity and pre-diabetes were enrolled. All participants were white. There were no Hispanic individuals in the exenatide group and two in the metformin group, which did not differ significantly (P = 0.490). As shown in Table 1, variables at baseline were similar between the groups. All but three tolerated the maximal dose of metformin (1000 mg, BID) and all but three tolerated the maximal dose of exenatide (10 mcg, BID). Medication compliance for both groups was excellent (metformin = 96 ± 5%; exenatide = 97 ± 4%).

**Table 1:** Baseline Characteristics

**Variable**	**Metformin Group(N = 25)**	**Exenatide Group(N = 25)**	**P-Value**
Age (years)	58.4 ± 10.1	58.7 ± 10.0	0.911
Gender (male/female)	7/18	5/20	0.742
BMI (kg/m^2^)	35.8 ± 7.0	35.3 ± 5.5	0.781
Waist Circumference (cm)	112.3 ± 15.7	111.6 ± 12.5	0.861
Body Fat (%)	44.5 ± 4.9	42.3 ± 6.5	0.264
SBP (mmHg)	125.8 ± 12.3	130.6 ± 17.1	0.260
DBP (mmHg)	75.6 ± 10.0	74.3 ± 9.3	0.620
Cholesterol (mg/dL)	185.0 ± 33.1	187.4 ± 28.7	0.782
LDL-Cholesterol (mg/dL)	101.9 ± 27.2	104.1 ± 26.9	0.777
HDL-Cholesterol (mg/dL)	53.2 ± 14.3	53.8 ± 11.9	0.864
Triglycerides (mg/dL)	137.2 ± 43.0	149.0 ± 58.4	0.419
Glucose (mg/dL)	103.1 ± 9.1	103.2 ± 9.9	0.976
Insulin (mU/L)	9.6 ± 8.1	8.5 ± 4.7	0.538
HOMA-IR	2.5 ± 2.4	2.2 ± 1.4	0.547
CRP (mg/L)	4.3 ± 2.2	4.9 ± 3.2	0.510
OxLDL (U/L)	142.8 ± 66.7	123.7 ± 61.4	0.301
VCAM-1 (ng/mL)	529.1 ± 178.6	495.5 ± 119.9	0.440
RHI	2.03 ± 0.66	2.03 ± 0.57	0.989

BMI = body mass index; SBP = systolic blood pressure; DBP = diastolic blood pressure; LDL = low density lipoprotein; HDL = high density lipoprotein; HOMA-IR = homeostasis model assessment for insulin resistance; CRP = C-reactive protein; OxLDL = oxidized low density lipoprotein; VCAM-1 = vascular cell adhesion molecule-1; RHI = reactive hyperemic index.

Body fat data were obtained in 35 patients (exenatide N = 17; metformin N = 18)

Data are shown as mean ± standard deviation.

**Figure 1:**
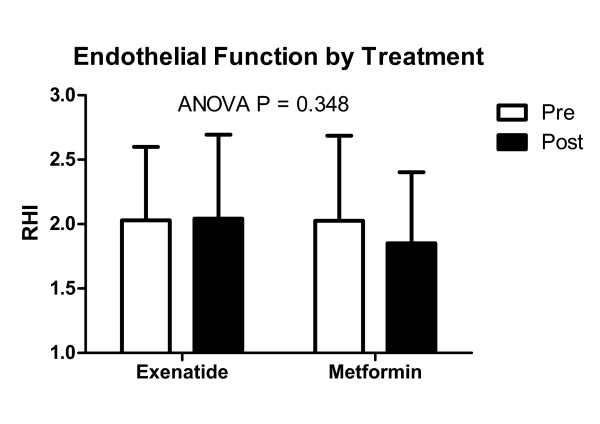
Endothelial function before and after 3-months of treatment with either exenatide or metformin.

Table 2 shows the treatment effects by group. There were no differences in the changes from baseline for RHI (Δ exenatide: 0.01 ± 0.68 vs. Δ metformin: -0.17 ± 0.72, P = 0.348) (Figure 1), CRP (Δ exenatide: -0.4 ± 2.2 mg/L vs. Δ metformin: -0.4 ± 2.2 mg/L, P = 0.987), oxLDL (Δ exenatide: -0.1 ± 41.5 U/L vs. Δ metformin: -16.5 ± 30.4 U/L, P = 0.123), or VCAM-1 (Δ exenatide: 10.4 ± 83.2 ng/mL vs. Δ metformin: -15.3 ± 101.3 ng/mL, P = 0.336) between exenatide and metformin groups. Triglycerides were reduced more with exenatide compared to metformin (Δ exenatide: -25.5 ± 45.7 mg/dL vs. Δ metformin: -2.9 ± 22.8 mg/dL, P = 0.032). There were no differences in any of the other variables including visceral (pre- and post-exenatide: 201.2 ± 44.4 cm^2^ to 206.3 ± 44.3 cm^2^, respectively vs. pre- and post-metformin: 286.6 ± 114.4 cm^2^ to 278.3 ± 122.8 cm^2^, P = 0.208) and subcutaneous (pre- and post-exenatide: 473.9 ± 152.5 cm^2^ to 457.8 ± 143.1 cm^2^, respectively vs. pre- and post-metformin: 352.6 ± 148.4 cm^2^ to 349.6 ± 131.4 cm^2^, P = 0.448) fat in the subset with computed tomography data (not presented in Table 2).

**Table 2:** Treatment Effects by Group

**Variable**	**Δ Metformin(N = 25)**	**Δ Exenatide(N = 25)**	**Difference(M – E)**	**P-Value**
BMI (kg/m^2^)	−0.4 ± 0.7*	−0.8 ± 1.4*	−0.4	0.229
Waist Circumference (cm)	−1.7 ± 3.5*	−3.2 ± 6.2*	−1.5	0.285
Body Fat (%)	0.8 ± 1.7	0.8 ± 2.6	0.0	0.939
SBP (mmHg)	0.5 ± 10.2	−5.4 ± 15.3	−5.9	0.114
DBP (mmHg)	−0.8 ± 6.9	−2.8 ± 7.3	−2.0	0.322
Cholesterol (mg/dL)	−9.2 ± 16.1**	−14.6 ± 17.4***	−5.4	0.264
LDL-Cholesterol (mg/dL)	−1.1 ± 21.4	−9.9 ± 22.5*	−8.8	0.169
HDL-Cholesterol (mg/dL)	−1.5 ± 9.7	−1.1 ± 12.8	0.4	0.901
Triglycerides (mg/dL)	−2.9 ± 22.8	−25.5 ± 45.7*	−22.6	0.032
Glucose (mg/dL)	−3.7 ± 9.3	−2.7 ± 9.9	1.0	0.725
Insulin (mU/L)	−2.4 ± 4.3**	−0.6 ± 2.8	1.8	0.079
HOMA-IR	−0.8 ± 1.4*	−0.2 ± 0.8	0.6	0.094
CRP (mg/L)	−0.4 ± 2.2	−0.4 ± 2.2	0.0	0.987
OxLDL (U/L)	−16.5 ± 30.4*	−0.1 ± 41.5	16.4	0.123
VCAM-1 (ng/mL)	−15.3 ± 101.3	10.4 ± 83.2	25.7	0.336
RHI	−0.17 ± 0.72	0.01 ± 0.68	0.18	0.348

BMI = body mass index; SBP = systolic blood pressure; DBP = diastolic blood pressure; LDL = low density lipoprotein; HDL = high density lipoprotein; HOMA-IR = homeostasis model assessment for insulin resistance; CRP = C-reactive protein; OxLDL = oxidized low density lipoprotein; VCAM-1 = vascular cell adhesion molecule-1; RHI = reactive hyperemic index.

Data are shown as mean ± standard deviation; P-Values represent ANOVA interaction term.

Body fat data were obtained in 35 patients (exenatide N = 17; metformin N = 18).

* denotes P<0.05 for within group treatment effect; ** denotes P<0.01 for within group treatment effect; *** denotes P<0.001 for within group treatment effect.

### Sub-study

**Figure 2:**
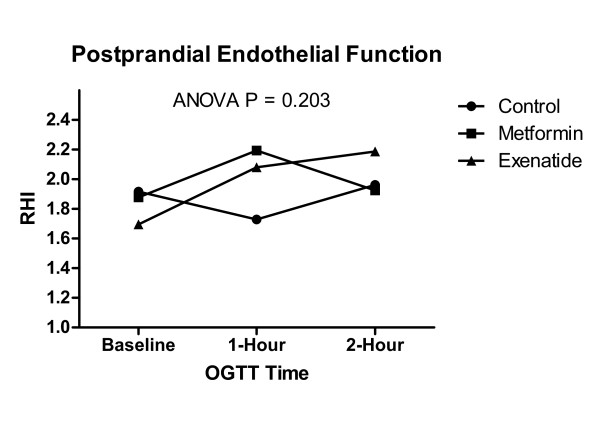
Serial endothelial function during the OGTT for control (no drug pre-administration) and pre-administration of either exenatide or metformin.

All participants (mean age 47.6 ± 12.8; 4 females, 3 males) in the sub-study had IGT. There was a trend toward a difference in glucose AUC during the OGTT among the three conditions (control AUC: 7,691 ± 2,925 units, vs. exenatide AUC: 4,382 ± 2,421 units, vs. metformin AUC: 5,859 ± 2,210 units, ANOVA P = 0.076) and no statistically significant difference in insulin AUC (control AUC: 5,535 ± 1,838 units, vs. exenatide AUC: 4,543 ± 2,389 units, vs. metformin AUC: 4,084 ± 1,800 units, ANOVA P = 0.410). There was no statistically significant difference in postprandial RHI AUC among the three conditions (control AUC: -10.0 ± 50.5 units, vs. exenatide AUC: 37.8 ± 51.1 units, vs. metformin AUC: 20.5 ± 47.5 units, ANOVA P = 0.203) (Figure 2).

## Discussion

Little is known about the effect of chronic GLP-1 receptor agonist treatment on endothelial function in humans. The primary finding of the current study is that chronic treatment with exenatide in patients with obesity and pre-diabetes has similar effects on microvascular endothelial function, inflammation, oxidative stress, and vascular activation as treatment with metformin. These results are in line with a recent pilot study we conducted in non-diabetic youth with severe obesity, which demonstrated no change in endothelial function with 3-months of exenatide treatment [29]. In regard to inflammation and oxidative stress, our results are in contrast to a recent study that demonstrated a rapid anti-inflammatory and reactive oxygen species suppression effect of exenatide in patients with T2DM [30]. The contradictory findings between these studies may be explained by the different patient populations examined (pre-diabetes vs. T2DM) and the methods by which inflammation and oxidative stress were measured (systemic vs. cellular/molecular). Two other studies have evaluated the effects of acute, one-time, GLP-1 administration on endothelial function in patients with IGT and T2DM [9,19]. Nystrom et al. [19] reported that infusion of GLP-1 in a small number (N = 12) of patients with T2DM and stable coronary artery disease improved brachial artery flow-mediated dilation while no improvement was observed in healthy controls. Koska et al. [9] demonstrated improved postprandial (high-fat meal) RHI with pre-administration of exenatide (10 mcg) in patients with either IGT or T2DM. Taken together, these findings suggest that improvements in endothelial function with GLP-1 receptor agonists may be limited to the postprandial setting.

There are many potential explanations for the findings in this study - the most obvious being that GLP-1 receptor agonists may have minimal or no appreciable effect on endothelial function and blood flow in individuals with obesity and pre-diabetes. The lack of improvement in endothelial function may be related to the relatively short half-life of exenatide (approximately 2.4 hours) and the fact that its metabolic effects occur predominantly in the postprandial setting. Alternatively, the treatment period may not have been long enough in this study to elicit an improvement in endothelial function. Some studies have shown that weight loss and glycemic control continue to improve well beyond 3-months with exenatide therapy [31-33]. Another potential explanation for the lack of improvement in endothelial function is that, despite the high baseline BMI, body fat, CRP, and other risk factor levels, the baseline RHI values in these patients were relatively high (2.03 in both groups), which may have limited the ability of exenatide to exert a beneficial effect. Therefore, it is possible that results may differ in other patient populations, such as those with T2DM who often have significant endothelial dysfunction. Finally, it is possible that the effects of exenatide on endothelial function may differ by vascular bed, and that the microvasculature, which was measured in this study, is less responsive compared to the conduit arteries.

Evidence exists supporting a role for GLP-1 receptor agonists having a beneficial effect on postprandial endothelial function. Koska et al. recently reported that pre-administration of exenatide significantly enhanced endothelial function following a high-fat meal in individuals with IGT or newly-diagnosed T2DM [9]. Reductions in postprandial triglycerides explained over 60% of the effect of exenatide on endothelial function improvement, suggesting that the beneficial postprandial vascular effects of GLP-1 receptor agonists are likely mediated via the lowering of triglycerides [9,34]. This may explain the lack of effect in postprandial endothelial function with exenatide observed in our study. Instead of a high-fat meal, which would be expected to raise postprandial triglycerides, we utilized a glucose-only meal. Although we did not measure postprandial triglycerides in the current study, it is unlikely that levels increased very much following the glucose load. Therefore, the current state of the evidence suggests that improvements in endothelial function with GLP-1 receptor agonists may be limited to the postprandial period (0–3 hours) primarily in relation to a high-fat meal.

Strengths of this study included the randomized/controlled design, the similarities between the exenatide and metformin groups at baseline, and the fact that subjects were not using any T2DM medications. Limitations included the non-blinded design, the lack of a non-treatment control group, and the small sample size for the sub-study. Because the mean improvement in postprandial endothelial function was higher with exenatide compared to metformin and control (albeit non-significantly), it is possible that significant differences may have been observed with a larger sample size.

In conclusion, 3-months of exenatide therapy had similar effects on microvascular endothelial function, markers of inflammation, oxidative stress, and vascular activation, as treatment with metformin, in patients with obesity and pre-diabetes. Improvements in endothelial function with GLP-1 receptor agonists may be limited to the postprandial setting, particularly following the consumption of a high-fat meal. Future studies should examine the vascular effects of combined treatment with a GLP-1 receptor agonist and metformin, evaluate the effects in patients with T2DM, utilize treatment periods longer than 3-months, and evaluate the other vascular beds (e.g., conduit arteries).

## Authors’ contributions

A.S.K. participated in study design, data collection, data analysis and interpretation, and drafted the manuscript; R.M.B. participated in data collection, data interpretation, and edited the manuscript; J.M.G.C. participated in data interpretation and edited the manuscript; H.K. participated in data interpretation and edited the manuscript; A.J.B. participated study design, data collection, data interpretation, and edited the manuscript.

## Disclosures

A.S.K. has received research support from GlaxoSmithKline and Amylin/Eli Lilly and is a consultant (clinical trials advisory board) for Novo Nordisk. R.M.B. receives research support from Amylin, Eli Lilly, Novo Nordisk, Sanofi, Boehringer Ingelheim, Merck, Roche and Takeda; consults with Amylin, Eli Lilly, Novo Nordisk, Sanofi, Roche and Takeda; he receives no personal income from any of these activities. J.M.G.C. receives research support from Novartis, Pfizer, Sanofi-Aventis, Novo Nordisk, Leptos, Takeda, Amylin, Astra-Zeneca, Boehringer Ingelheim, and Ipsen; is a member of the speaker’s bureau for Merck, Pfizer, Boehringer Ingelheim, Forest, and GlaxoSmithKline; and is a consultant for Merck, Pfizer, Leptos, and Roche. H.K. is a member of the speaker’s bureau for Eli Lilly, Novo Nordisk, and Amylin. A.J.B. is a member of the speaker’s bureau for Forest Pharmaceuticals and receives research support from GlaxoSmithKline and Amylin/Eli Lilly.

## Funding

Funding was provided by an investigator-initiated grant (awarded to A.S.K.) from Amylin Pharmaceuticals and Eli Lilly and Company. The funders were not involved in the data analysis or manuscript preparation.
